# Three-Dimensional Hydrogen-Bonded Porous Metal-Organic Framework for Natural Gas Separation with High Selectivity

**DOI:** 10.3390/molecules29020424

**Published:** 2024-01-15

**Authors:** Wenyan Dan, Guangfeng Wei, Xiangdong Fang

**Affiliations:** College of Chemical Science and Engineering, Tongji University, 1239 Siping Road, Yangpu, Shanghai 200092, China

**Keywords:** metal-organic frameworks, hydrogen bond, natural gas, separation

## Abstract

A 3D hydrogen-bonded metal-organic framework, [Cu(apc)_2_]_n_ (**TJU-Dan-5**, Hapc = 2-aminopyrimidine-5-carboxylic acid), was synthesized via a solvothermal reaction. The activated **TJU-Dan-5** with permanent porosity exhibits a moderate uptake of 1.52 wt% of hydrogen gas at 77 K. The appropriate BET surface areas and decoration of the internal polar pore surfaces with groups that form extensive hydrogen bonds offer a more favorable environment for selective C_2_H_6_ adsorption, with a predicted selectivity for C_2_H_6_/CH_4_ of around 101 in C_2_H_6_/CH_4_ (5:95, *v*/*v*) mixtures at 273 K under 100 kPa. The molecular model calculation demonstrates a C–H···π interaction and a van der Waals host–guest interaction of C_2_H_6_ with the pore walls. This work provides a strategy for the construction of 3D hydrogen-bonded MOFs, which may have great potential in the purification of natural gas.

## 1. Introduction

Metal-organic frameworks, as self-assembled porous materials, have been widely explored in the last two decades due to their excellent performance in the areas of gas storage and separation [1,2], heterogeneous catalysis [3], drug delivery [4], luminescence [5,6,7], electrochemistry [8], and magnetism [9]. The structure and properties of MOFs depend on the nature of their metal cations and bridging ligands. Designing MOFs with an eye for novel structures and utilities through the sagacious choice of various metal ions and ligands always constitutes one of the most intriguing research topics in chemistry and materials science [10,11,12]. Among the factors that may influence the structures of MOFs, hydrogen bonding is capable of providing overall structural rigidity and diversity [13], and the stability of the corresponding molecular networks can be enhanced by augmenting the number or strength of the hydrogen bonds in which each tectonic subunit participates [14]. However, hydrogen bonds are greatly affected by various factors, such as intermolecular distances, temperature, pressure, and solvents [15]. As a result, reports concerning the design and synthesis of functional MOFs involving both coordination bonds and hydrogen bonds are rather scarce in the chemical literature [16,17].

**Figure 1:**
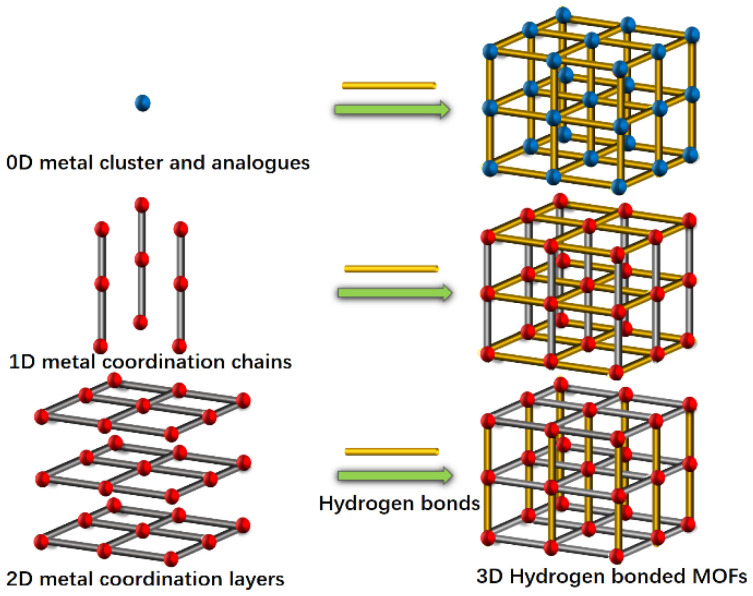
Schematic diagram showing the construction of 3D hydrogen-bonded MOFs in three ways (blue balls represent a metal cluster or macrocycle; the orange rods represent hydrogen bonds among ligands; red balls represent a metal or metal cluster; and the grey rods represent organic ligand parts).

Three-dimensional hydrogen-bonded MOFs are usually assembled based on coordinated hydrogen bond interactions. According to the structural characteristics of reported 3D hydrogen-bonded MOFs, they can be constructed in three ways (Figure 1). The first is that the metal and ligand form a 0D cluster including an MOP (metalorganic polyhedron) or a macrocycle including MOCs (metal-organic cubes) and MOSs (metalorganic squares), which are further linked together along three directions to generate 3D frameworks [18,19,20]. Eddaoudi and Liu reported some hydrogen-bonded MOFs made with MOCs and MOSs [21,22,23,24] containing 3D hydrogen-bonding interactions. In this respect, MOC-2 and MOC-3, consisting of indium (III) and 4,5-dicyanoimidazole, were constructed from vertex-to-vertex hydrogen-bonded MOCs [21]. The second is that 1D chains of metal and ligands are hydrogen-bonded along two orthogonal dimensions to give a 3D framework. Román synthesized a copper(II)-isophthalato MOF containing a 9-methyladenine nucleobase, exhibiting features of the second 3D hydrogen-bonded MOFs [25]. The last choice is similar to pillar-layered structures, in which the 2D networks of a metal and ligand are further interconnected by hydrogen bonds to generate 3D frameworks, as shown by {[Zn(apc)_2_]·H_2_O}_n_ and DAT-MOF-1 [26,27].

However, great synthetic challenges still exist in constructing 3D hydrogen-bonded MOFs due to the less tunable character of hydrogen bonds. Using preorganized 2D metal organic sheets assembled by hydrogen bonds may be an alternative strategy to solve the above morass, albeit encountering the quandary of selecting suitable metal centers as well as ligands.

With recent developments in MOFs and HOFs (hydrogen-bonded organic frameworks), the separation properties of MOF and HOF materials have attracted considerable research and commercial interest based on the conception of CO_2_ capture [28] and purification of natural gas [29], ethane [30], and butadiene [31]. In fact, the high porosities of materials are not prerequisites for their gas separation applications. As reported in the literature, MOFs and HOFs exhibiting excellent performance for gas separations are those of moderate porosities, with BET surface areas less than 1000 m^2^/g [32]. The design and synthesis of new porous MOF materials with hydrogen-bonding features may serve as a new concept to take advantage of the structural features of both materials with the aim of gas separation applications. Liu’s group reported some hydrogen-bonded MOFs like ZSA-7, ZSA-8, and ZSA-9. ZSA-7 and ZSA-8 exhibit high CO_2_ adsorption abilities (109.8 and 114.0 cm^3^·g^−1^ at 273 K, respectively, under 1 bar) and outstanding natural gas selectivity separation, especially for CO_2_ over CH_4_ (39.8 and 35.7 for CO_2_/CH_4_ = 0.5/0.5 and 76.0 and 51.6 for CO_2_/CH_4_ = 0.05/0.95, respectively, under 1 bar at 298 K) [33]. Natural gas is a hydrocarbon mixture primarily consisting of 85% CH_4_, 9% C_2_H_6_, 3% C_3_H_8_, 2% N_2_, and 1% C_4_H_10_. To reduce the percentage of ethane content, it is very important to purify natural gas. With CH_4_ as a premium choice for greenhouse effect reduction, using MOFs for the industrial separation of CH_4_ and C_2_H_6_ in natural gas becomes urgent and necessary. As a fruitful approach, selectivity enhancements in gas separation have been successfully carried out through the precise control of the pore size and environment of MOFs [34]. However, the selectivity of C_2_H_6_ and CH_4_ is low in the reported MOFs, and it may be due to the inappropriate BET surface areas and weak interactions of C_2_H_6_ with the cavity surface of the MOF frameworks [30,32]. In this respect, 3D hydrogen-bonded MOFs may be an excellent choice of porous materials for CH_4_/C_2_H_6_ purification of natural gas.

Based on our preliminary study [26], we herein used the aminopyrimidylcarboxylate ligand (2-aminopyrimidine-5-carboxylic acid) and Cu(NO_3_)_2_ to deliver a 3D hydrogen bonded framework (namely **TJU-Dan-5**) through a solvothermal reaction, which consists of 2D **sql** networks connected by hydrogen bonds. The 1D permanent porosity of **TJU-Dan-5** exhibits higher C_2_H_6_/CH_4_ selectivity (101:1, at 273 K/100 kPa) due to C–H···π interactions between C_2_H_6_ and the pore wall. In addition, **TJU-Dan-5** displays a high selectivity for equimolar C_2_H_6_/C_2_H_4_ mixture gas (selectivity: 2.46 at 298 K/100 kPa). This work demonstrates that internal hydrogen bonding implanted in the pore of MOFs provides both enhanced structural integrity and C_2_H_6_ friendly pore surfaces. 

## 2. Results and Discussion

### 2.1. Structure Description

The crystallographic data and structure refinement information are listed in Table S1. **TJU-Dan-5**, Cu(apc)_2_, is crystallized in the monoclinic space group of *C*2/*c*. The asymmetric unit consists of one crystallographically independent Cu^2+^ ion and two molecules of apc^−^ ligand (Figure S1). As shown in Figure S2, the Cu^2+^ ion coordinates four carboxylate oxygen (Cu–O, 1.976(4) Å and 1.972(4), Table S2) and one pyrimidine nitrogen atom (Cu–N, 2.177(4) Å) has a distorted tetragonal pyramid conformation. The Hapc carboxylate groups coordinate the copper ion in a bidentate fashion (Figure S2), with the C–O bond distances of the carboxylate group ranging from 1.254(6) Å to 1.265(6) Å. Compared with the reported structure [26], Hapc adopted new coordination modes (III and IV, Figure S3). One apc ligand connects three copper ions through two Cu–O bonds and one Cu–N bond (mode IV, Figure S3). The other one apc ligand chelates two copper ions through two Cu–O bonds, and the aminopyrimidine group does not coordinate with copper ion (mode III, Figure S3). The free aminopyrimidine forms a hydrogen bond between amino and carboxylate instead (*d*_N–H···N_ = 2.988(6) Å, Figure S3 and Table S3). It is noteworthy that internal hydrogen bonds exist within aminopyrimidine groups (*d*_N–H···N_ = 2.984(6) Å, 3.051(7) Å, 3.054(6) Å, Figure S4 and Table S3). 

**Figure 2:**
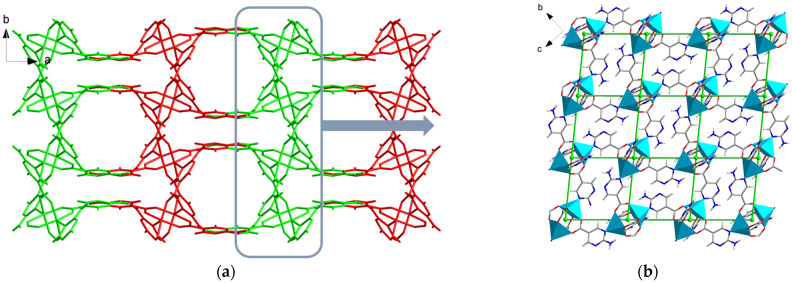
Structure of **TJU-Dan-5**. (**a**) Packing diagram of **TJU-Dan-5** along *c* axis, (red: one two-dimensional coordination layer; green: the other two-dimensional coordination layers). (**b**) The two-dimensional layer can be simplified as **sql** topology (green grid) along *a* axis.

**Figure 3:**
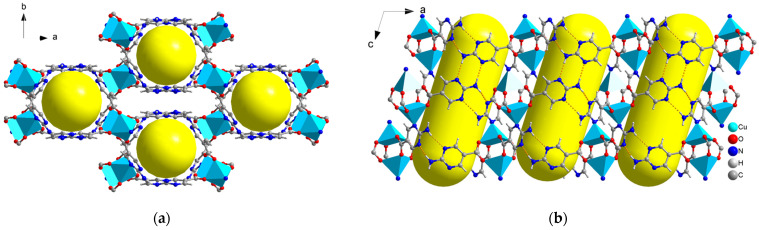
Packing view of **TJU-Dan-5**. (**a**) Yellow balls are added to highlight the porosity along c axis, (**b**) yellow columns are added to highlight the 1D channels, along *b* axis (C: dark gray; N: dark blue; O: red; H: light gray, Cu_2_(CO_2_)_2_N_2_: two light blue tetrahedrons. Hydrogen bonds within aminopyrimidine groups are represented in orange dotted lines.

**Figure 4:**
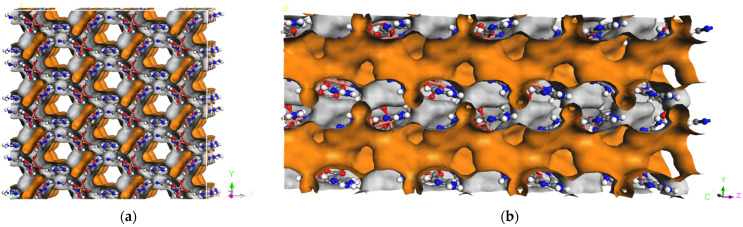
Connolly surface area (orange/grey curved surface) calculated with probe atomic radii of 1.4 Å in **TJU-Dan-5**. (**a**) Along *Z* axis, (**b**) along *X* axis.

The Cu paddle-wheel SBU(Cu_2_(CO_2_)_2_N_2_) produced by two CuO_4_N_1_ tetrahedrons are corner-shared to form a 2D network along the *a* axis (Figure 2a, green). The other 2D network (Figure 2b, red) is also composed of Cu paddle-wheel SBU and a 1D helix chain along the *b* axis. A similar pillared-layer 3D framework is generated by internal hydrogen bonds among apc ligands of the 2D network (Figure 1, resulting in a one-dimensional hexagonal channel with the pore size of 5.0 Å × 6.0 Å by the van der Waals radius along *c* axis (Figure 3). The void volume is 1272.9 Å^3^ (36.5%) as estimated by PLATON using a probe of 1.2 Å and the Connolly surface area is shown in Figure 4. Usually, 4, 4′-Bipyridine or analogue linker usually bridges the porous pillared-layer frameworks through coordination bonds [35]. However, the internal hydrogen bonds within aminopyrimidine groups link the 2D layers to construct the 3D porous pillar-layer network of **TJU-Dan-5**, the structure type rarely reported in MOFs. In contrast to most MOFs with metal ions or clusters coordinating with organic ligands, the framework of **TJU-Dan-5** provides a new example of 3D hydrogen-bonded MOFs (Figure 1). It is thus interesting to further explore the topology analysis of such hydrogen-bonded frameworks for the rational design of reticular chemistry of MOFs.

**Figure 5:**
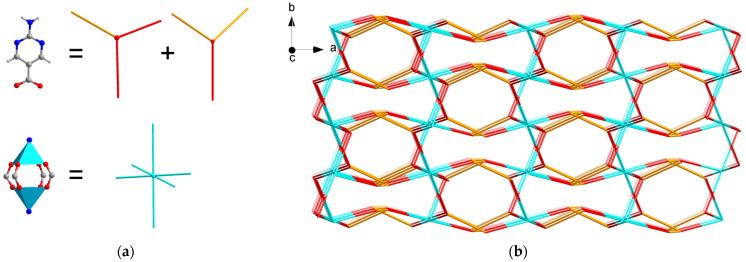
The simplified node and topology of **TJU-Dan-5**: (**a**) the simplified apc^−^ ligands in two 3-connected nodes (orange lines represent hydrogen bonds) and the simplified Cu paddle-wheel SBU Cu_2_(CO_2_)_2_N_2_ in 6-connected node; (**b**) the network of **TJU-Dan-5** showing in (3,3,6)-connected new topology.

The topology of **TJU-Dan-5** was analyzed by using the TOPOSPro program [36]. Firstly, the two-dimensional network formed by the metal cluster and ligands was investigated. Considering Cu paddle-wheel SBU(Cu_2_(CO_2_)_2_N_2_) as a 4-connected node, each layer yields a 2D 4-connected uninodal net with point symbol of {4^4^.6^2^}, which is a **sql** net (Figure 2b). Furthermore, hydrogen bonds participating in the 3D framework of **TJU-Dan-5** should be considered in the process of analyzing the topology. The two crystallographically independent apc^−^ ligands with different hydrogen bonds are regarded as two 3-connected nodes (Figure 5a), Cu paddle-wheel SBU(Cu_2_(CO_2_)_2_N_2_) as a 6-connected node, and the total 3-D network displays a trinodal (3,3,6)-connected net with point (Schläfli) symbol {6^3^}_4_{6^6^.8^4^.10^5^}. According to the search results from the Reticular Chemistry Structure Resource (RCSR) database and the literature, only seven trinodal (3,3,6)-connected nets have been reported, including the symbol names of **brk**, **muo**, **tsa**, **tsy**, **xbq**, **zxc** [37], and another one with point symbols of {4^2^.6}{4^3^}{4^5^.6^4^.8^6^} [38]. Therefore, **TJU-Dan-5** provides a completely new topology in MOF crystal nets.

### 2.2. Characterization of **TJU-Dan-5**

The thermal stability of **TJU-Dan-5** was further evaluated via a thermogravimetric analysis (TGA) and varied-temperature PXRD. A sharp weight loss above 270 °C in the TGA curve corresponded to the departure of the organic ligands and the collapse of the framework (Figure S5), while the varied-temperature PXRD patterns obtained at increasing temperatures show that the framework collapses above 270 °C (Figure S6). The framework of **TJU-Dan-5**, like most MOFs based Cu-paddle-wheel SBU, is stable up to 250 °C in air, both suggesting that hydrogen-bonded MOFs materials are structurally robust.

### 2.3. Gas Adsorption of H_2_ and N_2_

Adsorption experiments were thus carried out to evaluate the rigidity and permanent porosity of **TJU-Dan-5**. The N_2_ sorption at 77 K for activated **TJU-Dan-5** shows type I adsorption isothermal behavior, characteristic of a microporous material with permanent porosity (Figures S7 and S8). The Brunauer–Emmett–Teller (BET) and Langmuir surface area of **TJU-Dan-5** were determined to be 748.7 m^2^·g^−1^ and 914.2 m^2^·g^−1^, respectively. The experimental pore volume was calculated to be 0.323 cm^3^·g^−1^. The pore size distribution of **TJU-Dan-5**, analyzed by the NLDFT model, displays a peak of6.0 Å in the micropore region, which is close to the size of the hexagonal channel in the crystal structure of title compound (Figure S7, inset). Hydrogen adsorption reveals a moderate uptake of 1.52 wt% at 77 K (1 atm) (Figure S9). **TJU-Dan-5** may be studied as a potential hydrogen storage material. The *Q*st (H_2_) is slightly lower than that of MOF-74(Zn) but considerably higher than those of other porous materials, such as MOF-177, JUC-48, and PCN-17 [39]. The isosteric heat of H_2_ sorption, calculated by fitting the adsorption data at 77 K and 87 K to the Virial equation, was found to be 7.98 kJ/mol at zero coverage (Figures S10 and S11).

### 2.4. Separations of C_2_H_6_/CH_4_ and C_2_H_6_/C_2_H_4_

**Figure 6:**
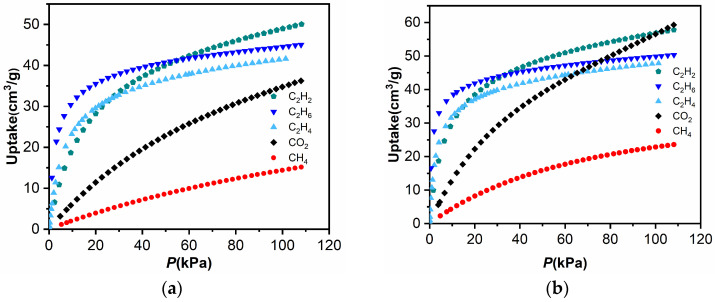
CO_2_, CH_4_, C_2_H_2_, C_2_H_4_, and C_2_H_6_ adsorption isotherms for **TJU-Dan-5** measured at 298 K (**a**) and 273 K (**b**).

**Figure 7:**
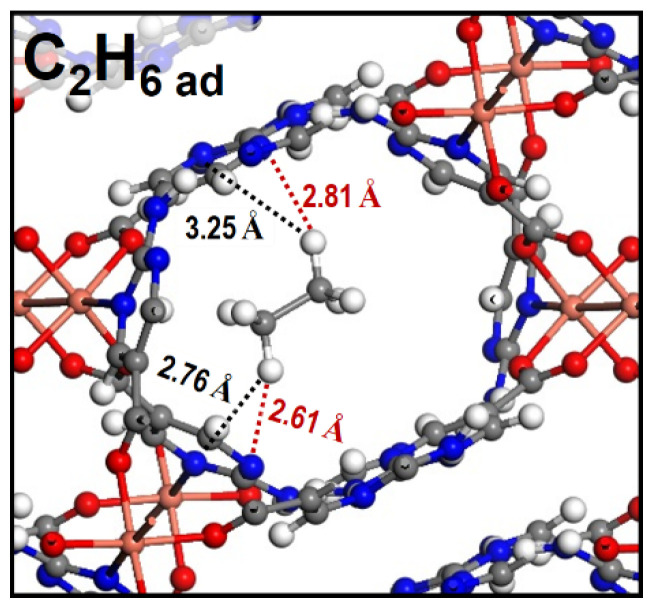
DFT-calculated optimized C_2_H_6_ adsorption site, van de Waals host-guest interactions (red dotted lines), and C–H···π interaction (black dotted lines) C_2_H_6_ and **TJU-Dan-5**. Color codes: Cu, pink; C, gray; H, white; O, red; and N, blue.

Separation of C_2_H_6_ from natural gas is crucial in sufficient utilization of natural gas and CO_2_ reduction in the earth atmosphere. The unique channel sizes of **TJU-Dan-5** us to investigate its potential in the C_2_H_6_/CH_4_ separation from natural gas. In this respect, single-component sorption isotherms of CH_4_ and C_2_H_6_ were measured at 273 K and 298 K, respectively. For **TJU-Dan-5**, the absorbed amounts of CH_4_ at 273 and 298 K are only 23.6 and 15.2 cm^3^·g^−1^, respectively (Figure 6), whereas the corresponding C_2_H_6_ amounts of gas uptake at 273 and 298 K are 50.3 and 45.03 cm^3^·g^−1^(Figure 6). More importantly, under low pressure below 30 kPa, **TJU-Dan-5** takes up much more C_2_H_6_ than CH_4_. **TJU-Dan-5** takes up a much different amount of C_2_H_6_ and CH_4_, suggesting of separation selectivity of **TJU-Dan-5** in C_2_H_6_ and CH_4_. In addition, C_2_H_2_, C_2_H_4_, and CO_2_ gas sorption isotherms of **TJU-Dan-5** were measured at 273 K and 298 K, respectively (Figure 7). **TJU-Dan-5** can take up the amounts of 57.79 cm^3^·g^−1^ and 50.06 cm^3^·g^−1^ for C_2_H_2_, 47.86, 41.64 cm^3^·g^−1^ and 36.25 cm^3^·g^−1^ for C_2_H_4_, and 59.27 cm^3^·g^−1^ and 36.25 cm^3^·g^−1^ for CO_2_ at different temperature levels (Figure 4). 

To obtain binding energy at low coverage, isosteric adsorption heats of C_2_H_6_, CH_4_, C_2_H_2_, and C_2_H_4_ for **TJU-Dan-5** were calculated through the Virial equation and using the Clausius–Clapeyron relation, respectively (Figures S12 and S13). As shown in Figure S12, the isosteric adsorption heat of C_2_H_6_ is higher than the others. At zero coverage of C_2_H_6_ interaction with the most energetically favored adsorption sites, the enthalpy of 35.5 kJ mol^−1^ can be attributed to stronger van der Waals host–guest interactions and C–H···π interaction between **TJU-Dan-5** and C_2_H_6_. Pyridine rings presented in **TJU-Dan-5** may bring C_2_H_6_ in close contact with the pore walls. To visualize the preferential binding sites of C_2_H_6_, DFT-based structural potential energy surface searching identified strong C–H···π interactions between C_2_H_6_ and the framework (the distances between H and pyrimidine centroids: 2.76 and 3.25 Å in Figure 7) and van der Waals host–guest interaction (the nearest distances between H and pyrimidine: 2.61 and 2.81 Å in Figure 7). In fact, the calculated adsorption energy of C_2_H_6_ on **TJU-Dan-5** is 34.7 kJ/mol, in an excellent agreement with the experimental data.

**Figure 8:**
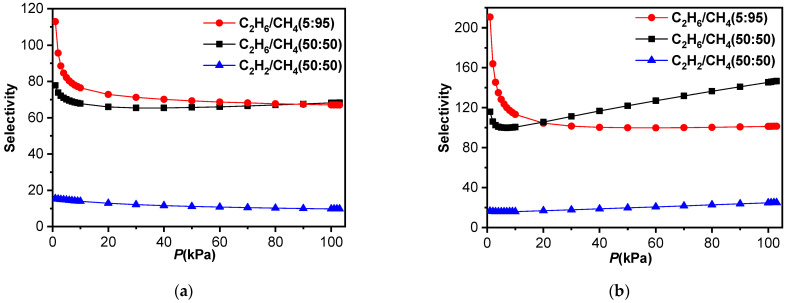
Selectivity predicted via DSLF-IAST method for adsorption of equimolar binary mixture (C_2_H_2_/CH_4_, CH_4_/C_2_H_6_) in TJU-Dan-5 at 298 K (**a**) and 273 K (**b**).

**Figure 9:**
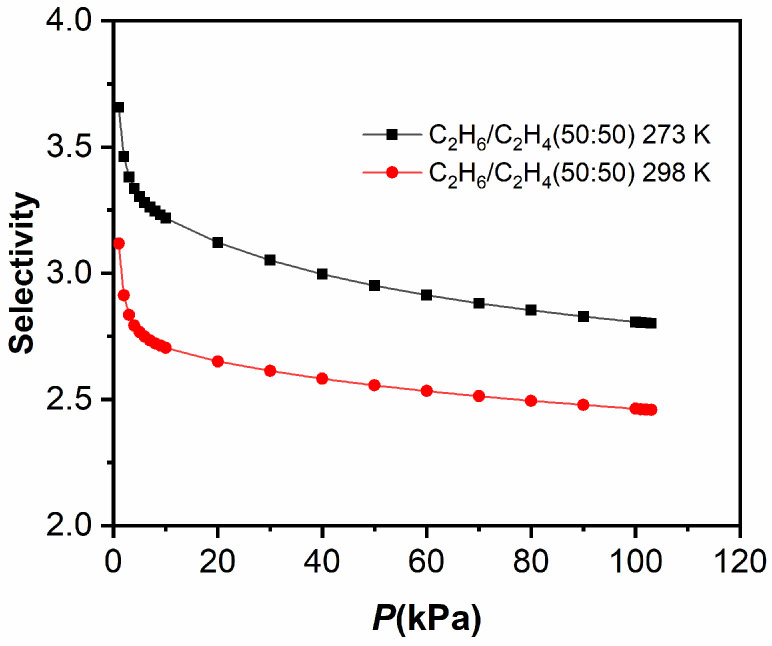
Selectivity predicted via DSLF-IAST method for adsorption of equimolar binary mixture C_2_H_6_/C_2_H_4_ in **TJU-Dan-5** at 298 K and 273 K.

The ideal adsorbed solution theory (IAST) is used to calculate C_2_H_6_/CH_4_, C_2_H_6_/C_2_H_4_, C_2_H_2_/CH_4_ [40] and gas mixture selectivity respectively (see the Supporting information, Equations (S1) and (S2)). **TJU-Dan-5** shows the selectivity of C_2_H_6_/CH_4_ (68 at 50:50), C_2_H_6_/CH_4_ (67 at 5:95), 298 K (100 kPa); C_2_H_6_/CH_4_ (146 at 50:50), C_2_H_6_/CH_4_ (101 at 5:95), 273 K (100 kPa); C_2_H_2_/CH_4_ (15 at 50:50), 298 K (100 kPa); C_2_H_2_/CH_4_ (25at 50:50), 273 K (100 kPa) (Figure 8). In the calculation, **TJU-Dan-5** exhibits greater separation ratios of C_2_H_6_/CH_4_ at both 273 K and 298 K. The high selectivities of **TJU-Dan-5** in C_2_H_6_/CH_4_ separation are better than those of MOFs or HOFs (Table 1) reported at room temperature, such as FJI-C4 [41], UTSA-33 [42], SNNU-Bai67 [43], VNU-18 [44], SBMOF-2, and HOF-BTB [45,46]. These results may be attributed to the stronger affinity between the pore environment of **TJU-Dan-5** and C_2_H_6_, making **TJU-Dan-5** a good choice for natural gas purification. In addition, it is worth noting that the selectivity for C_2_H_6_/C_2_H_4_ equimolar mixtures is 2.46 at 298 K (100 kPa) (Figure 9), only lower than some leading MOFs in gas separation, such as MAF-49 (2.9) [30], Zn-FBA (2.9) [47], Cu(Qc)_2_ (3.4) [48], and Fe_2_(O_2_)(dobdc) (4.4) [49], supporting our prediction of **TJU-Dan-5** utilization in gas separation.

**Figure 10:**
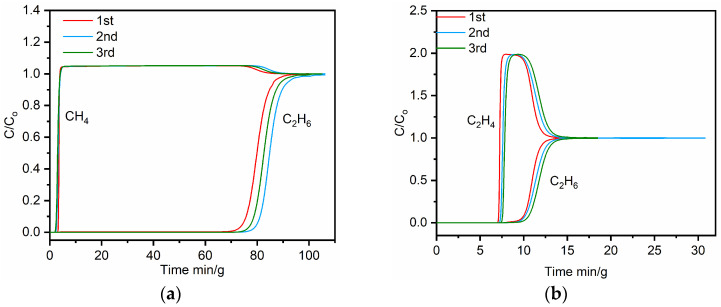
Experimental column breakthrough curves of **TJU-Dan-5**. (**a**) CH_4_/C_2_H_6_ (5/95) mixture under a flow of 5 mL·min^−1^, (**b**) C_2_H_4_/C_2_H_6_ (50/50) mixture under a flow of 5 mL·min^−1^ in an absorber bed packed with **TJU-Dan-5** at 298 K and 1.0 bar. (The results were individually tested three times).

Dynamic breakthrough experiments toward C_2_H_6_/CH_4_ (5:95, *v*/*v*) and C_2_H_6_/C_2_H_4_ (50:50, *v*/*v*) were carried out at 298 K (Figure 10). The activated **TJU-Dan-5** sample was packed into a column, with the binary gas mixtures of CH_4_/C_2_H_6_ and C_2_H_4_/C_2_H_6_ flowing at 5.0 mL min^−1^ at room temperature. As shown in Figure 10a, CH_4_ was immediately monitored in the outlet of the fixed bed, while the C_2_H_6_ was retained in the column packed with **TJU-Dan-5** for 70 min/g. The results demonstrate that **TJU-Dan-5** is capable of trapping C_2_H_6_ molecules from CH_4_/C_2_H_6_ mixtures. High-purity CH_4_ products (≥99.99%) can be directly obtained, and during the time of 0 to 66 min/g (C_2_H_6_ does not reach its breakthrough point), the amounts of CH_4_ captured in **TJU-Dan-5** were calculated to be 299.31 mL/g. In Figure 10b, C_2_H_4_ was eluted from a column before C_2_H_6_ with different retention times of 7.5 and 10.3 min·g^−1^, respectively, in an equimolar mixture. The purity of CH_4_ was determined to be ≥99.04%. The breakthrough curves are kept over three cycles, indicating that the sample is sufficiently stable at the given conditions. In addition, the PXRD experiments further confirm the working stability of **TJU-Dan-5** following the sorption experiments and breakthrough tests (Figure S14). Separation of absolute C_2_H_6_ from CH_4_/C_2_H_6_ mixture can be achieved by using activated **TJU-Dan-5** under ambient conditions. 

## 3. Materials and Methods

### 3.1. General Naterials and Methods

All reagents for syntheses were purchased from commercial sources. Thermogravimetric (TGA) analyses were investigated with a Mettler Toledo TGA/SDTA851 analyzer (Mettler Toledo, Zurich, Switzerland) in N_2_ atmosphere with a heating rate of 5 K min^−1^, from 30 °C to 800 °C. Elemental analyses (C, N, H) were measured on an Elementar Vario EL III microanalyzer (Elementar, Frankfurt, Germany). IR spectra were measured from a KBr pellets on a Thermo Scientific Nicolet IS10 FT-IR spectrometer (Thermo Scientific, Waltham, MA, USA) in the range of 4000–400 cm^−1^. Powder X-ray diffraction (PXRD) patterns were carried out using a Bruker D8 powder diffractometer (Bruker, Karlsruhe, Germany) at 40 kV, 40 mA for Cu Kα radiation (λ = 1.5406 Å), with a scan speed of 0.2 s/step and a step size of 0.05° (2θ).

### 3.2. Synthesis of **TJU-Dan-5**

[Cu(apc)_2_]_n_ (**TJU-Dan-5**, Hapc = 2-aminopyrimidine-5-carboxylic acid) was obtained via solvothermal synthesis. A mixture of Hapc (0.014 g, 0.1 mmol), Cu(NO_3_)_2_·3H_2_O (0.013 g, 0.06 mmol), DMF (2.0 mL), and anhydrous methyl alcohol (0.5 mL) was placed in a 10 mL glass bottle and stirred for 1 h at room temperature. After the mixture was sealed in a Pyrex tube and heated at 60 °C for 2 days, the whole apparatus was cooled to room temperature. Blue plate crystals of **TJU-Dan-5** were collected via filtration (yield: 55% based on Cu(NO_3_)_2_·3H_2_O). Elemental analysis (%): calcd. for [Cu(apc)_2_]_n_ (339.75): C 35.35, H 2.37, N 24.74; found: C 35.41, H 2.50, N 24.60. IR (KBr, cm^−1^): 3401 cm^−1^ (m), 3303 cm^−1^ (w), 1686 cm^−1^ (w), 1646 cm^−1^ (w), 1601 cm^−1^ (m), 1508 cm^−1^ (w), 1414 cm^−1^ (m), 1357 cm^−1^ (m), 1246 cm^−1^ (w), 1181 cm^−1^ (w), 1084 cm^−1^ (m), 1003 cm^−1^ (w), 844 cm^−1^ (m), 810 cm^−1^ (w), 683 cm^−1^ (w), 602 cm^−1^ (w), 477 cm^−1^ (m).

### 3.3. X-ray Crystallography

The data for **TJU-Dan-5** were collected from a single crystal at 296(2) K on a Bruker D8 VENTURE dual-wavelength Mo/Cu three-circle diffractometer with a microfocus sealed X-ray tube using mirror optics as monochromator and a Bruker PHOTON II detector (Bruker, Karlsruhe, Germany). MoKα radiation (λ = 0.71073 Å) was MoKα used in the diffractometer. All data were integrated with SAINT and a multi-scan absorption correction using SADABS was applied [57,58]. The structure was solved by direct methods using SHELXT and refined by full-matrix least-squares methods against *F*^2^ by SHELXL-2019/1 [59,60]. All non-hydrogen atoms were refined with anisotropic displacement parameters. The hydrogen atoms were refined isotropically on calculated positions using a riding model with their Uiso values constrained to 1.5 times the Ueq of their pivot atoms for terminal sp^3^ carbon atoms and 1.2 times for nitrogen and all other carbon atoms. The crystallographic data for the structures reported in this paper have been deposited with the Cambridge Crystallographic Data Centre [61]. CCDC **1830585** for **TJU-Dan-5** includes the supplementary crystallographic data for this paper. These data can be acquired free of charge via www.ccdc.cam.ac.uk/data_request/cif (accessed on 21 April 2020), or by emailing data_request@ccdc.cam.ac.uk, or by contacting The Cambridge Crystallographic Data Centre, 12, Union Road, Cambridge CB2 1EZ, UK; fax: +44 1223 336033. This report and the CIF file were generated using FinalCif [62]. 

### 3.4. Gas Adsorption Measurements

The as-synthesized materials of **TJU-Dan-5** were washed with DMF and CH_3_OH for three times, respectively. Solvent was exchanged with CH_2_Cl_2_ for nine times over three days. CH_2_Cl_2_ was further removed with supercritical liquid CO_2_ in a Tousimis Samdri PVT-30 critical point dryer (Tousimis, Rockville, MD, USA). N_2_ adsorption and desorption measurements were measured on a Quantachrome Autosorb-iQ gas adsorption analyzer (Quantachrome, Tallahassee, FL, USA) and pore size analyzer at 77 K. The pore size distribution of **TJU-Dan-5** was analyzed by the NLDFT model utilizing N_2_ adsorption data at 77 K (calculation model: N_2_ at 77 K on carbon (slit/cylinder. pore, NLDFT equilibrium model); Eff. mol. Diameter (D): 3.54 Å). Before the gas adsorption tests, the experimental sample was immersed in CH_3_OH for 36 h, and then the exchanged sample was activated under vacuum at 100 °C for 10 h. Single H_2_ sorption experiments were measured on a Micromeritics ASAP 2020 adsorption analyzer (Micromeritics, Atlanta, GA, USA) at 77 K and 87 K via liquid N_2_ bath and liquid Ar bath, respectively. C_2_H_6_, C_2_H_2_, C_2_H_4_, CH_4_ and CO_2_ absorption experiments were performed on the Micromeritics ASAP 2020 adsorption analyzer at 273 K and 298 K via an ice-water bath and heating jacket, respectively. Two different temperatures isotherms were fitted to the Virial model and the isosteric heats of C_2_H_6_, C_2_H_4_, C_2_H_2_, and CH_4_ adsorption were calculated, respectively. The selectivity of C_2_H_6_ and C_2_H_2_ over CH_4_ was calculated with the ideal adsorbed solution theory (IAST) model (Equations (S1) and (S2)) at 273 K and 298 K.

### 3.5. Breakthrough Measurements

The breakthrough experiment was carried out using a multi-constituent adsorption breakthrough curve analyzer (BSD-MAB, Beishide Instrument Technology (Beijing) Co., Ltd., No. 607, Building 1, Brilliant International, Shangdi 10th Street, Haidian District, Beijing, China). The gas separation properties of **TJU-Dan-5** (1.26 g) were examined by breakthrough experiments using 0.05 (C_2_H_6_): 0.95 (CH_4_) and 0.5 (C_2_H_6_): 0.5 (C_2_H_4_) gas mixtures flowing through the activated samples packed into the same glass column (6.0 mm inner diameter, 65 mm in length), respectively. The gas mixture passed through the column at a rate of 5 mL/min. The composition of the effluent gas was detected by a Mass spectrometry. 

### 3.6. Density Functional Theory Calculations

Spin polarized density functional theory (DFT) calculations were performed using VASP packages with projected augmented wave (PAW) pseudo-potentials [63,64,65]. The exchange–correlation energy was treated based on the generalized gradient approximation (GGA) by using Perdew–Burke–Ernzerhof (PBE) functional [66]. The plane-wave cutoff energy was set to 450 eV. Brillouin zone sampling was restricted to the Gamma point [67]. The DFT-D3(BJ) method of Grimme were employed to describe long-range vdW interactions [68,69]. In this work, the crystal structure of the **TJU-Dan-5** framework was obtained according to the experimental characterization results. The optimized lattice parameters of **TJU-Dan-5** are 11.13 Å × 13.34 Å × 13.16 Å, with α = 106.74°, β = 90.01°, γ = 65.39°, which are close to the experimental characterization results. The preferential binding sites of small molecules were searched via the stochastic surface walking (SSW) method [70,71], which has been implemented in a computing program (LASP www.lasphub.com (accessed on 21 July 2022)). LASP is now available on market, and it can interface with VASP for all the functionalities. All atoms were fully relaxed during the lattice optimization. The Quasi-Newton l-BFGS method is used for geometry relaxation until the maximal force on each degree of freedom is less than 0.05 eV/Å.

## 4. Conclusions

A new 3D hydrogen bonding MOF (**TJU-Dan-5**) was successfully synthesized and characterized. As observed in the solid-state structure, two 2D metal coordination planes of **TJU-Dan-5** were ligated by hydrogen bonds, resulting in a 1D porous channel. Albeit no open metal site in **TJU-Dan-5**, the permanent porosity of **TJU-Dan-5** results in moderate performance for adsorption of H_2_ gas as well as high C_2_H_6_/CH_4_ and C_2_H_6_/C_2_H_4_ selectivity. The molecular model calculation result reveals strong C–H···π interactions between C_2_H_6_ and the pore wall, suggesting intermolecular hydrogen bonding in favor of pore integrity and C_2_H_6_ selection. We are exploring the possibility of diversifying the 3D hydrogen-bonded MOFs family for gas separation and further developing this synthetic concept.

## Figures and Tables

**Table 1:** Comparison of some typical MOFs and HOF for the separation of C_2_H_6_/CH_4_ and C_2_H_2_/CH_4_ as predicted by IAST.

MOF	BET	C_2_H_6_/CH_4_ (50:50)	C_2_H_6_/CH_4_ (5:95)	C_2_H_2_/CH_4_ (50:50)	T (K)	Gas Uptake C_2_H_6_ & CH_4_	Refs.
**TJU-Dan-5** *	748	146.5	101.2	25.0	273	50.3/23.6	This work
		68	67	15.4	298	45.03/15.2	This work
UTSA-35 *	742.7	15	/	19	296	51/9	[50]
[Co_2_(5,4-PMIA)_2_(TPOM)_0.5_]	920	34.7	/	32.0	300	81.9/16.9	[51]
FJI-C4 *	690	39.7	/	51	298	66.3/18.4	[41]
UTSA-33 *	660	20	/	17.1	296	59/12	[42]
Cu-TDPAT	1938	16.4	/	154.3	273	217.7/51.6	[52]
		12.1	/	127.1	298	154.4/28.3	[52]
JLU-MOF112 *	1553	12	24	/	298	107.7/10.2	[29]
SNNU-Bai67	989.5	38.3	/	50.5	298	91.2/15.8	[43]
VNU-18	900	27.3	/	41.5	298	72.8/18.5	[44]
SBMOF-2	195	26	/	18	298	59.8/16.2	[45]
ZJNU-119 *	950	20.9	/	62.9	298	89.2/37.5	[53]
HOF-BTBa	955	17.7	/	12.5	273	95.4/13.44	[46]
		13.7		9.3	295	69.2/10.08	[46]
PFC-5 *	256	19	/	15	298	25.9/8.0	[54]
HOF-14	2573	6.3		3.7	298	44.2/7.8	[55]
MAF-49		170	/	/	316	36/22	[30]
[Zn_2_(bdc)_2_(bpndi)]	565	175	/	496	298	44/8	[56]

* The selectivity value at 1 bar.

## Data Availability

Data are contained within the article and Supplementary Material.

## Supplementary Materials

The following supporting information can be downloaded at: https://www.mdpi.com/article/10.3390/molecules29020424/s1, Figure S1: The asymmetric unit of compound **TJU-Dan-5**; Figure S2: The coordinated environment around the metal center of **TJU-Dan-5**; Figure S3: The coordination mode of Hapc; Figure S4: Hydrogen bonds in **TJU-Dan-5**; Figure S5: TGA curve of active **TJU-Dan-5**; Figure S6: XRD patterns of **TJU-Dan-5** in air from room temperature to 270 °C; Figure S7: Nitrogen sorption isotherm of **TJU-Dan-5** at 77 K; Figure S8: BET surface area plot for **TJU-Dan-5**; Figure S9: Hydrogen sorption isotherm of **TJU-Dan-5**; Figure S10: Isosteric heat of adsorption *Q*st of H_2_ for **TJU-Dan-5**; Figure S11: The H_2_ isotherms at 77 K and 87 K and the Virial equation fits for **TJU-Dan-5**; Figure S12: Isosteric heat of adsorption *Q*st of C_2_H_6_, C_2_H_4_, C_2_H_2_, CH_4_ for **TJU-Dan-5**; Figure S13: The gas isotherms at 273 K and 298 K and the Virial equation fits for **TJU-Dan-5**; Figure S14: PXRD patterns of **TJU-Dan-5** after sorption experiments and breakthrough tests; Table S1: Crystal data and structure refinement of **TJU-Dan-5**; Table S2: Selected bond lengths (Å) and angles (º) for **TJU-Dan-5**; Table S3: Hydrogen bonds for **TJU-Dan-5**; Table S4: Parameters for DSLF isotherm fits at 298 K.
