# Supplementary material for: Three-Dimensional Hydrogen-Bonded Porous Metal-Organic Framework for Natural Gas Separation with High Selectivity
Source: Molecules. 2024 Jan 15;29(2):424. doi: 10.3390/molecules29020424 (PMC10820768; doi:10.3390/molecules29020424)
Supplement: Supplementary file 1 [file molecules-29-00424-s001.zip › molecules-2790003-supplementary.pdf]

*molecules*

# Supporting Information

## 3D Hydrogen-Bonded Porous Metal-Organic Framework for Natural Gas Separation with High Selectivity

Wenyan Dan*, Guangfeng Wei, and Xiangdong Fang*

*College of Chemical Science and Engineering, Tongji University, Siping Road 1239, Shanghai 200092, China.*

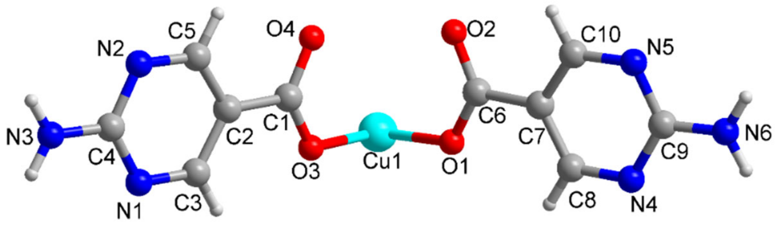


**Figure S1.** The asymmetric unit of compound **TJU-Dan-5**.

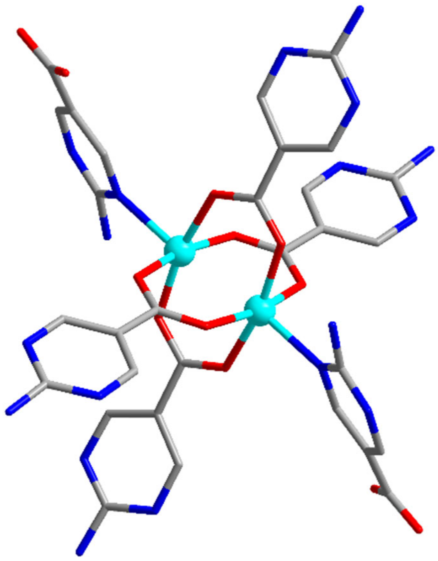

**Figure S2.** The coordinated environment around the metal center of **TJU-Dan-5** (Color code; Carbon: grey, oxygen: red, nitrogen: blue, copper: blue).

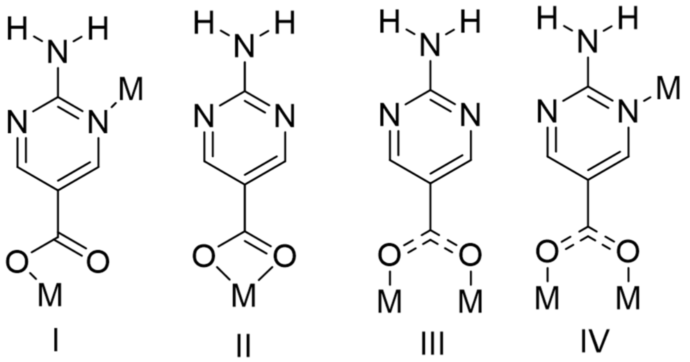


**Figure S3.** The coordination mode of Hapc.

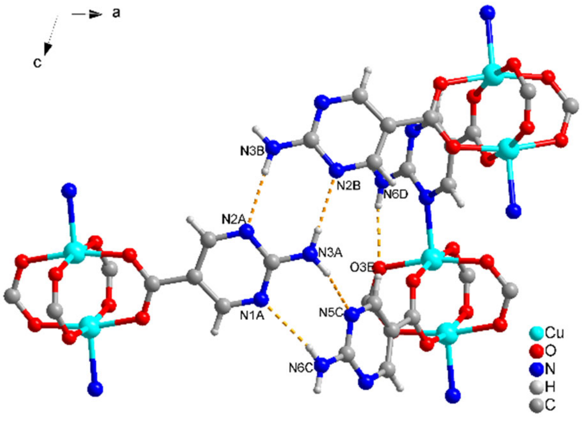


**Figure S4.** Hydrogen bonds in **TJU-Dan-5** (Symmetry codes: A: 1.5-x, 1.5-y, 1-z; B: 0.5+x, 1.5-y, -0.5+z; C: 2-x, 1-y, 1-z; D: 2-x, y, 0.5-z; E: 0.5+x, -0.5+y, z; orange dotted lines are added to highlight the Hydrogen bonds).

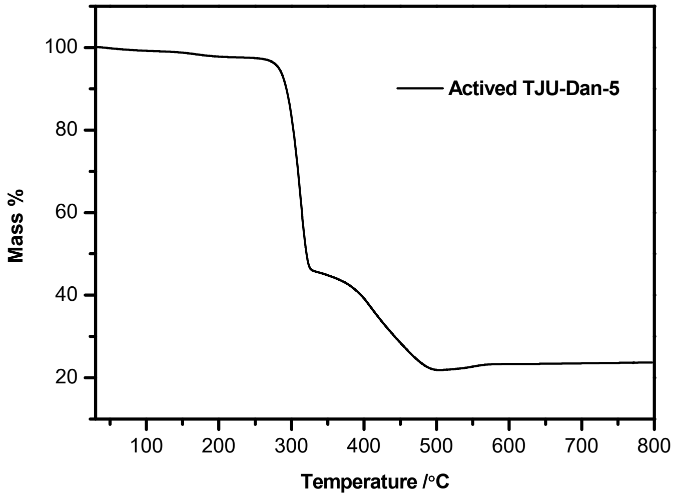


**Figure S5.** TGA curve of active **TJU-Dan-5**.

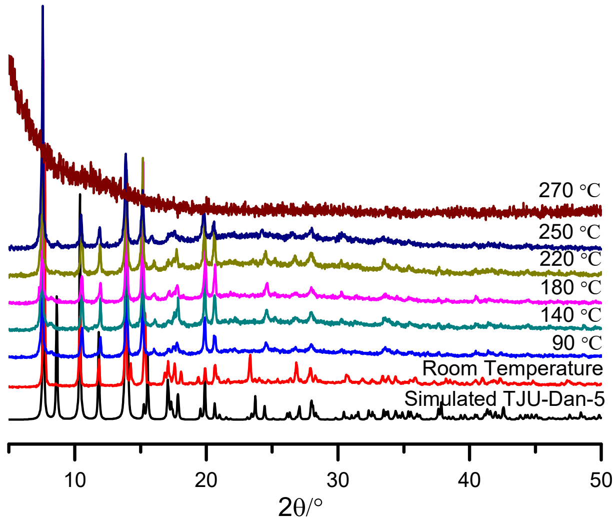


**Figure S6.** PXRD patterns of **TJU-Dan-5** in air from room temperature to 270 °C.

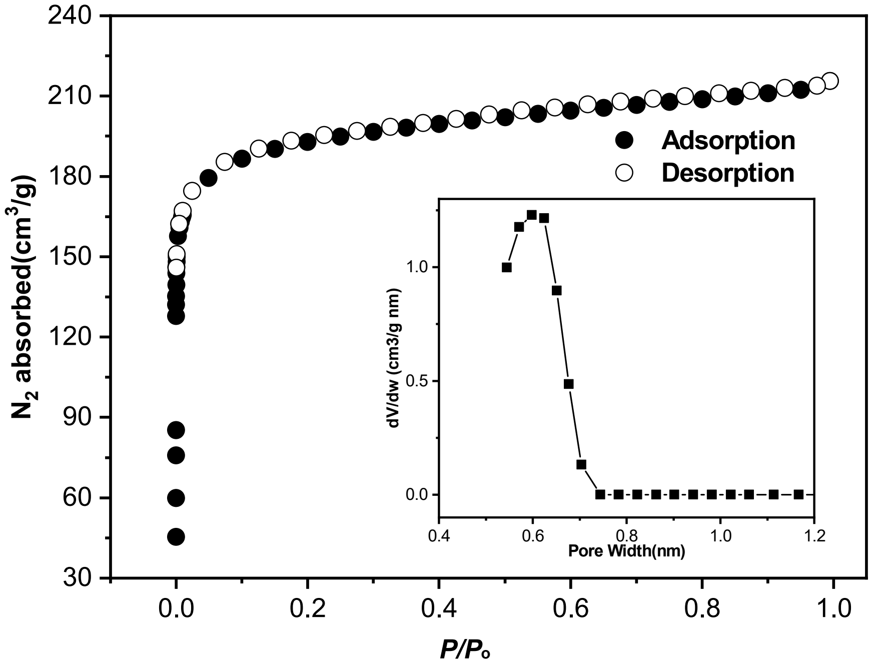


**Figure S7.** Nitrogen sorption isotherm of **TJU-Dan-5** at 77 K. The inset graph indicates the pore size distribution.

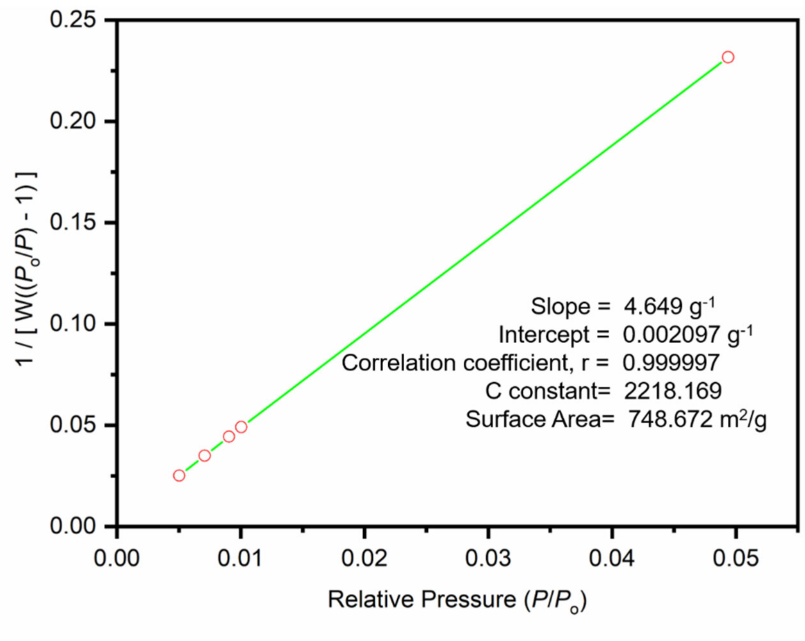


**Figure S8.** BET surface area plot for **TJU-Dan-5**

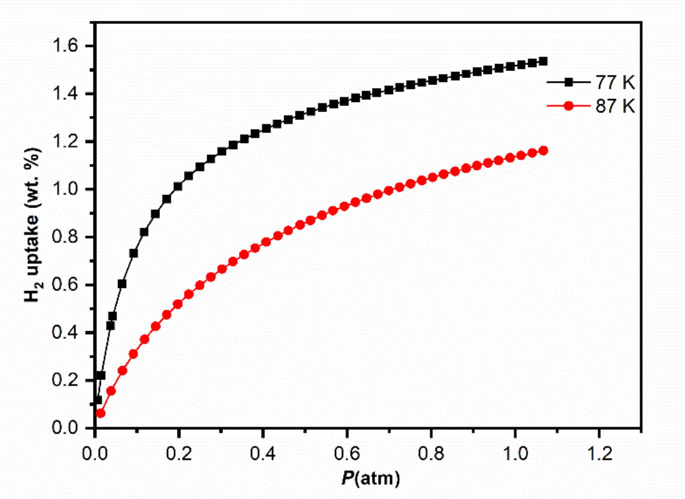


**Figure S9.** Hydrogen sorption isotherm of **TJU-Dan-5** at 77 K and 87 K.

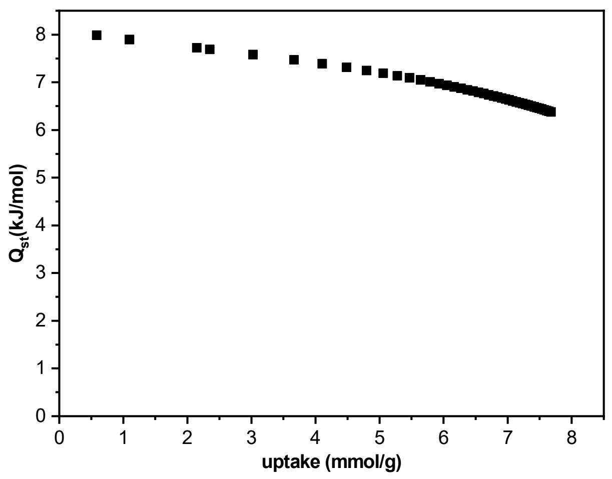


**Figure S10.** Isosteric heat of adsorption *Qst* of $H_2$ for **TJU-Dan-5**.

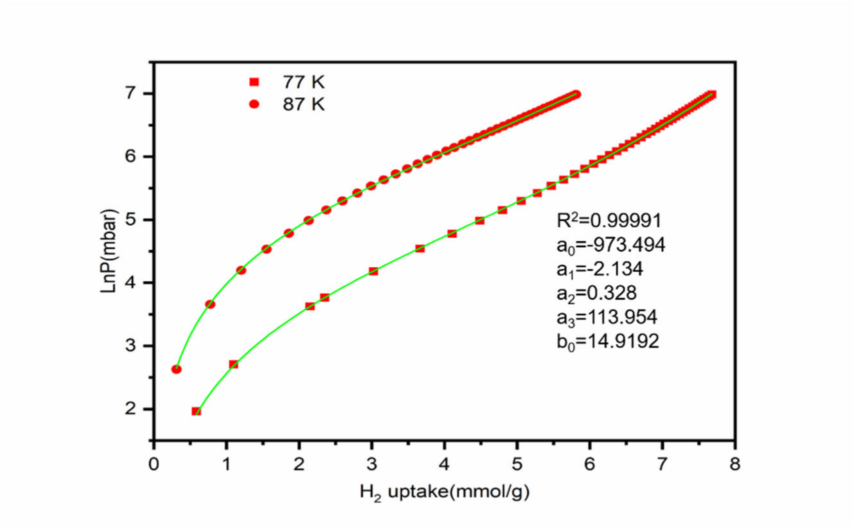


**Figure S11.** The $H_2$ isotherms at 77 K and 87 K (symbols) and the Virial equation fits (lines) for **TJU-Dan-5**

To perform the IAST calculations, an adsorption model is needed in order to fit a discrete set of adsorption data from single-component isotherms with a continuous function. Herein, we used a Dual-site Langmuir-Freundlich (DSLF, Equation (S1)) model for the fitting of the adsorption isotherms.

$$q = \frac{a \cdot b \cdot P^{c}}{1 + b \cdot P^{c}} + \frac{q \cdot k \cdot P^{t}}{1 + q \cdot P^{t}} \quad \text{(S1)}$$

*P* is the pressure of the bulk gas at equilibrium with the adsorbed phase (kPa), q is the adsorbed amount per mass of adsorbent (mmol/g), *a* and *q* are the saturation capacities of sites 1 and 2 (mmol/g), *b* and *k* are the

affinity coefficients of sites 1 and 2 (1/kPa), and *c* and *t* represent the deviations from an ideal homogeneous surface. R is the universal gas constant.

The adsorption selectivity for binary mixtures defined by Equation (S2).

$$S_{i/j} = \frac{x_i/x_j}{y_i/y_j} \qquad \text{(S2)}$$

where *S* is the selectivity factor, $x_i$ represents the quantity adsorbed of component *i,* and $y_i$ represents the partial pressure of component *i*.

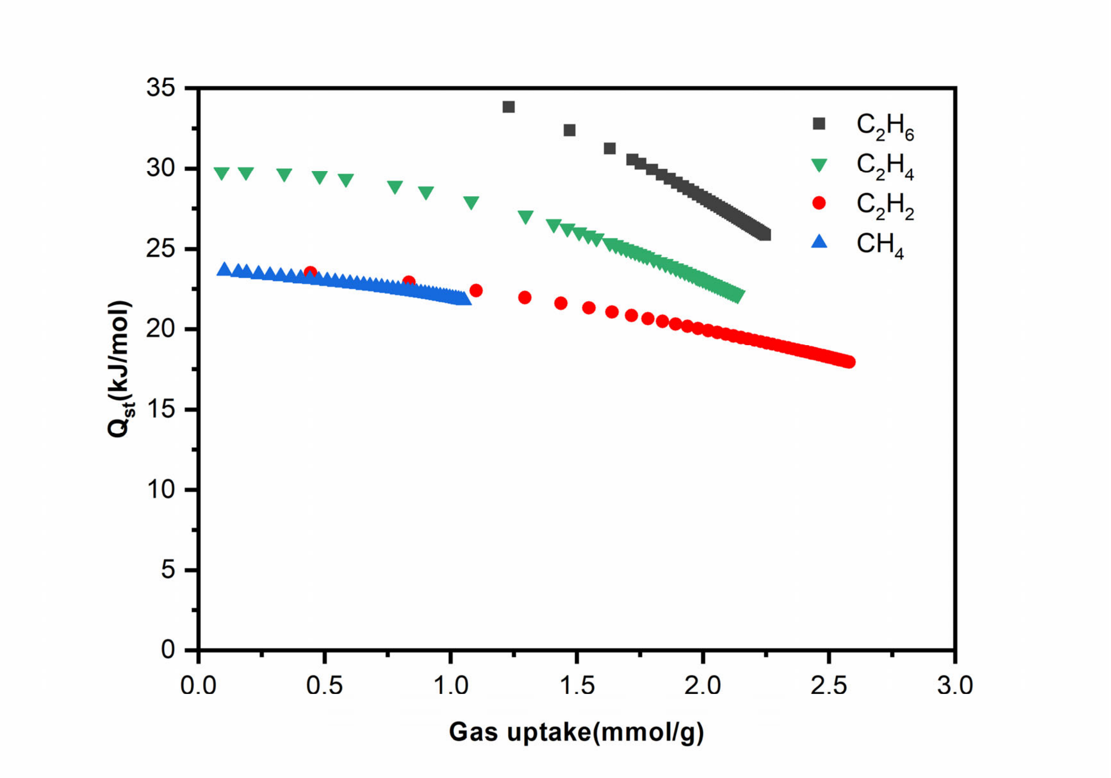


**Figure S12.** Isosteric heat of adsorption *Q*st of $C_2H_6$, $C_2H_4$, $C_2H_2$, $CH_4$ for **TJU-Dan-5**.

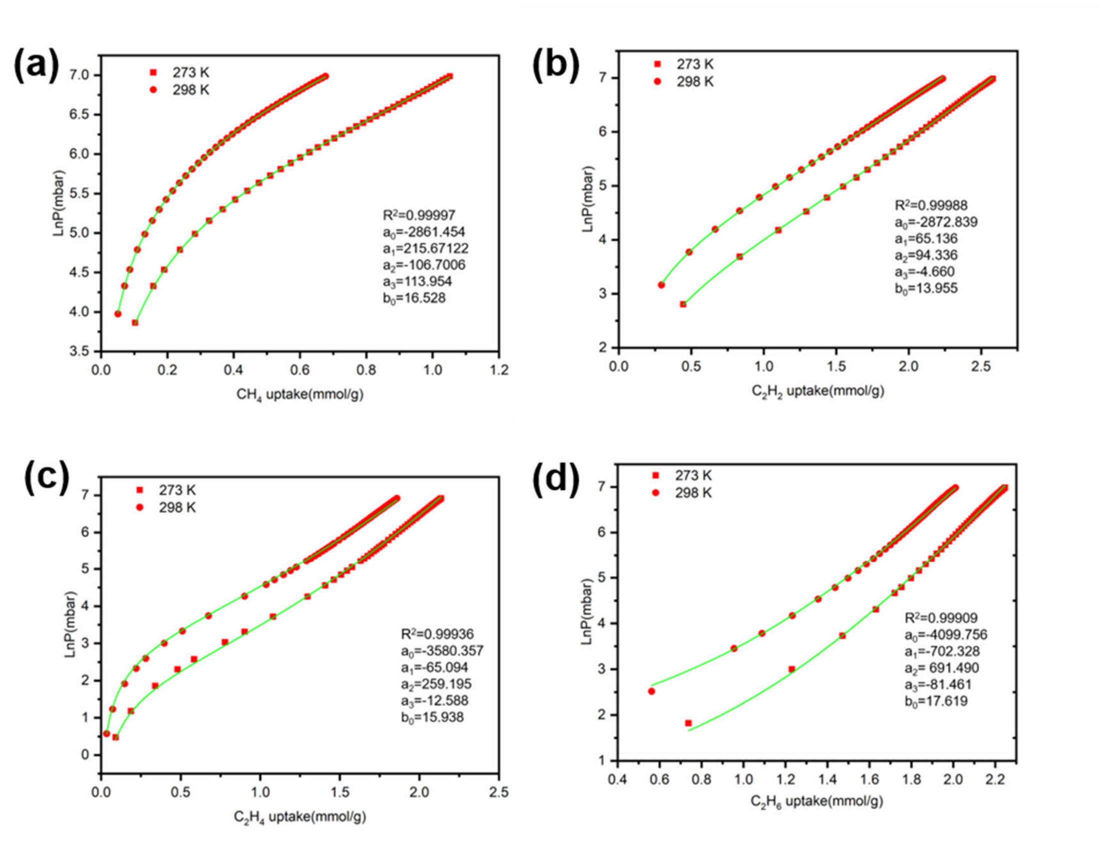


**Figure S13.** The gas isotherms at 273 K and 298 K (symbols) and the Virial equation fits (lines) for **TJU-Dan-5**: (a) $CH_4$, (b)$C_2H_2$, (c) $C_2H_4$, (d)$C_2H_6$.

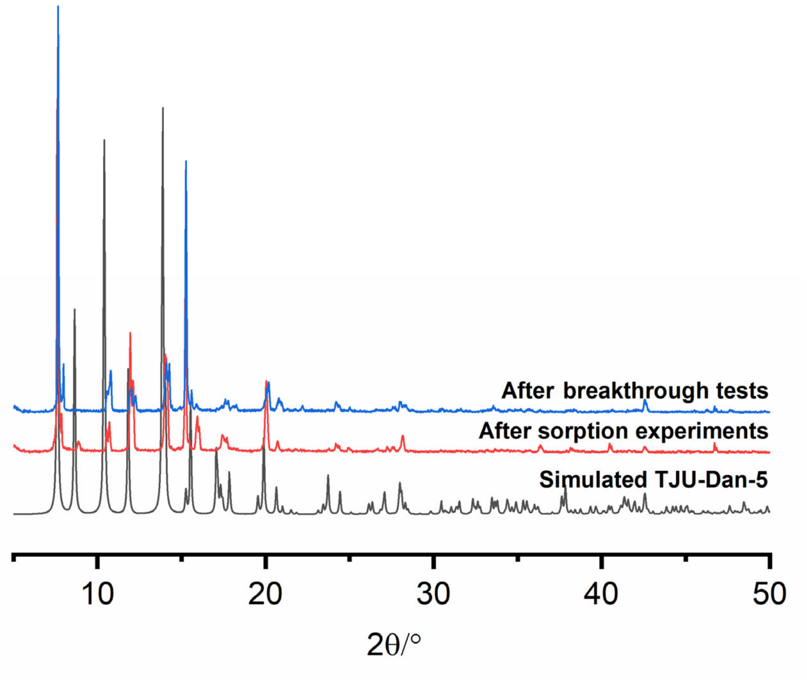


**Figure S14.** PXRD patterns of **TJU-Dan-5** after sorption experiments and breakthrough tests.

**Table S1.** Crystal data and structure refinement of **TJU-Dan-5**.

| Entry | **TJU-Dan-5** |
|---|---|
| Empirical formula | $C_{10}H_8CuN_6O_4$ |
| Formula weight | 339.75 |
| Temperature [K] | 296(2) |
| Crystal system | monoclinic |
| Space group (number) | $C2/c$ (15) |
| $a$ [Å] | 24.306(6) |
| $b$ [Å] | 11.394(3) |
| $c$ [Å] | 13.189(3) |
| α [°] | 90 |
| β [°] | 107.218(7) |
| γ [°] | 90 |
| Volume [Å$^3$] | 3489.1(15) |
| $Z$ | 8 |
| $\rho_{calc}$ [g·cm$^{-3}$] | 1.294 |
| $\mu$ [mm$^{-1}$] | 1.272 |
| $F$(000) | 1368 |
| Crystal size [mm$^3$] | 0.200×0.120×0.030 |
| Crystal colour | blue |
| Crystal shape | plate |
| Radiation | Mo$K_\alpha$ (λ=0.71073 Å) |
| 2Θ range [°] | 6.47 to 51.46 (0.82 Å) |
| Index ranges | $-29 \le h \le 29$ |
| | $-13 \le k \le 13$ |
| | $-16 \le l \le 16$ |
| Reflections collected | 28193 |
| Independent reflections | 3277 |
| $R_{int}$ | 0.0646 |
| $R_{sigma}$ | 0.0379 |
| Completeness to Θ = 25.242° | 99.6 % |
| Data / Restraints / Parameters | 3277/0/190 |
| Goodness-of-fit on $F^2$ | 1.059 |
| Final $R$ indexes | $R_1$ = 0.0555 |
| [$I$≥2σ($I$)] | w$R_2$ = 0.1544 |
| Final $R$ indexes | $R_1$ = 0.0674 |
| [all data] | w$R_2$ = 0.1623 |
| Largest peak/hole [e·Å$^{-3}$] | 1.12/-0.47 |

**Table S2.** Selected bond lengths (Å) and angles (º) for **TJU-Dan-5**.

| **TJU-Dan-5** | | | |
|---|---|---|---|
| Cu1–O3 | 1.972(4) | Cu1–O1 | 1.976(4) |
| Cu1–O2#1 | 1.973(4) | Cu1–N4#2 | 2.177(4) |
| Cu1–O4#1 | 1.975(4) | C1–O4 | 1.258(6) |
| O1–C6 | 1.264(6) | C1–O3 | 1.265(6) |
| N1–C3 | 1.314(7) | C1–C2 | 1.485(7) |
| N1–C4 | 1.348(7) | C2–C3 | 1.397(7) |
| O2–C6 | 1.254(6) | N3–C4 | 1.322(7) |
| N2–C5 | 1.328(7) | N5–C9 | 1.356(7) |
| N2–C4 | 1.356(7) | C6–C7 | 1.494(7) |
| C2–C5 | 1.375(7) | N6–C9 | 1.334(7) |
| N4–C8 | 1.341(6) | C7–C10 | 1.399(8) |
| N4–C9 | 1.357(7) | N5–C10 | 1.322(8) |
| O3–Cu1–O2 | 88.92(18) | C6–O1–Cu1 | 123.1(3) |
| O3–Cu1–O4 | 168.06(14) | C6–O2–Cu1 | 122.7(4) |
| O2–Cu1–O4 | 90.49(18) | C1–O3–Cu1 | 120.0(3) |
| O3–Cu1–O1 | 89.24(18) | C1–O4–Cu1 | 125.9(3) |
| O2–Cu1–O1 | 168.04(15) | C8–N4–Cu1 | 116.8(3) |
| O4–Cu1–O1 | 88.87(18) | C9–N4–Cu1 | 125.4(3) |
| O3–Cu1–N4 | 99.74(15) | O4–Cu1–N4 | 92.17(15) |
| O2–Cu1–N4 | 96.63(16) | O1–Cu1–N4 | 95.33(16) |

**Table S3.** Hydrogen bonds for **TJU-Dan-5**.

| **D–H···A [Å]** | **d(D–H) [Å]** | **d(H···A) [Å]** | **d(D···A) [Å]** | **<(DHA) [°]** |
|---|---|---|---|---|
| N3–H3A···N5#1 | 0.86 | 2.32 | 3.051(7) | 142.5 |
| N3–H3B···N2#2 | 0.86 | 2.13 | 2.984(6) | 169.5 |
| N6–H6B···O3#3 | 0.86 | 2.18 | 2.988(6) | 155.3 |
| N6–H6A···N1#4 | 0.86 | 2.33 | 3.054(6) | 142.3 |

Symmetry transformations used to generate equivalent atoms:
#1: -0.5+X, 0.5+Y, +Z; #2: 1-X, +Y, 1.5-Z; #3: 1.5-X, -0.5+Y, 0.5-Z; #4: 0.5+X, -0.5+Y, +Z;

**Table S4.** Parameters for DSLF isotherm fits at 298 K.

| | a | b | c | q | k | t | $R^2$ |
|---|---|---|---|---|---|---|---|
| **$CH_4$@TJU-Dan-5** | 0.07245 | 0.05856 | 0.97662 | 1.66511 | 0.00324 | 1.10952 | 0.9999 |
| **$C_2H_2$@TJU-Dan-5** | 1.10086 | 0.13390 | 1 | 1.81175 | 0.001683 | 1 | 0.9988 |
| **$C_2H_4$@TJU-Dan-5** | 1.40516 | 0.16663 | 1.09161 | 1.15993 | 0.01219 | 0.89703 | 1 |
| **$C_2H_6$@TJU-Dan-5** | 1.26825 | 0.43525 | 1.10229 | 1.59914 | 0.06749 | 0.55141 | 0.9999 |
